# Supplementary material for: Enhanced ice sheet melting driven by volcanic eruptions during the last deglaciation
Source: Nat Commun. 2017 Oct 24;8:1020. doi: 10.1038/s41467-017-01273-1 (PMC5654763; doi:10.1038/s41467-017-01273-1)
Supplement: Supplementary file 1 — Supplementary Information [file 41467_2017_1273_MOESM1_ESM.pdf]

Supplementary Table 1 – Melting event and corresponding volcanic eruptions. Age of exceptionally thick varve years (ETV) and corresponding volcanic sulfate anomalies in GISP2 ice cores. Events that are accompanied by acidity peaks in NGRIP electrical conductivity data (ecm) profiles are also reported.

Age ETV (GICC05 years BP)	Age from Vedde Ash (years)	Age sulfate peak (GICC05 years BP)	Age from Vedde Ash (years)	Max cum. Error (GICC05)	NGRIP2 ecm peak
12449	328	–	–	–	–
12475	354	12474	353	11	No
12550	429	12551	430	13	No
12551	430	12551	430	13	Yes
12565	444	12566	445	13	No
12571	450	12570	449	13	No
12617	496	12617	496	15	No
12618	497	12620	499	15	No
12683	562	–	–	–	–
12692	571	12693	572	17	No
12717	596	12715	594	18	No
12739	618	–	–	–	–
12831	710	12829	708	21	Yes
12847	726	12847	726	22	Yes
12981	860	12981	860	26	Yes
12992	871	12990	869	26	No
13060	939	13061	940	28	No
13114	993	–	–	–	Yes

Supplementary Table 2 – Summary of tephra horizons identified in Greenland ice cores. Tephra horizons identified during the period 13,200-12,300 GICC05 years BP that correspond to volcanic sulfate peaks in GISP2 records.

Core	Depth (m)	Age (GICC05 years BP)	Error	Name	Composition	Reference
NGRIP	1525.88	12830	n.a.	Unknown	R	1
NGRIP	1531.92	12980	70	Hekla	Intermediate (B, R)	1

n.a. not available
B: basaltic
R: rhyolitic

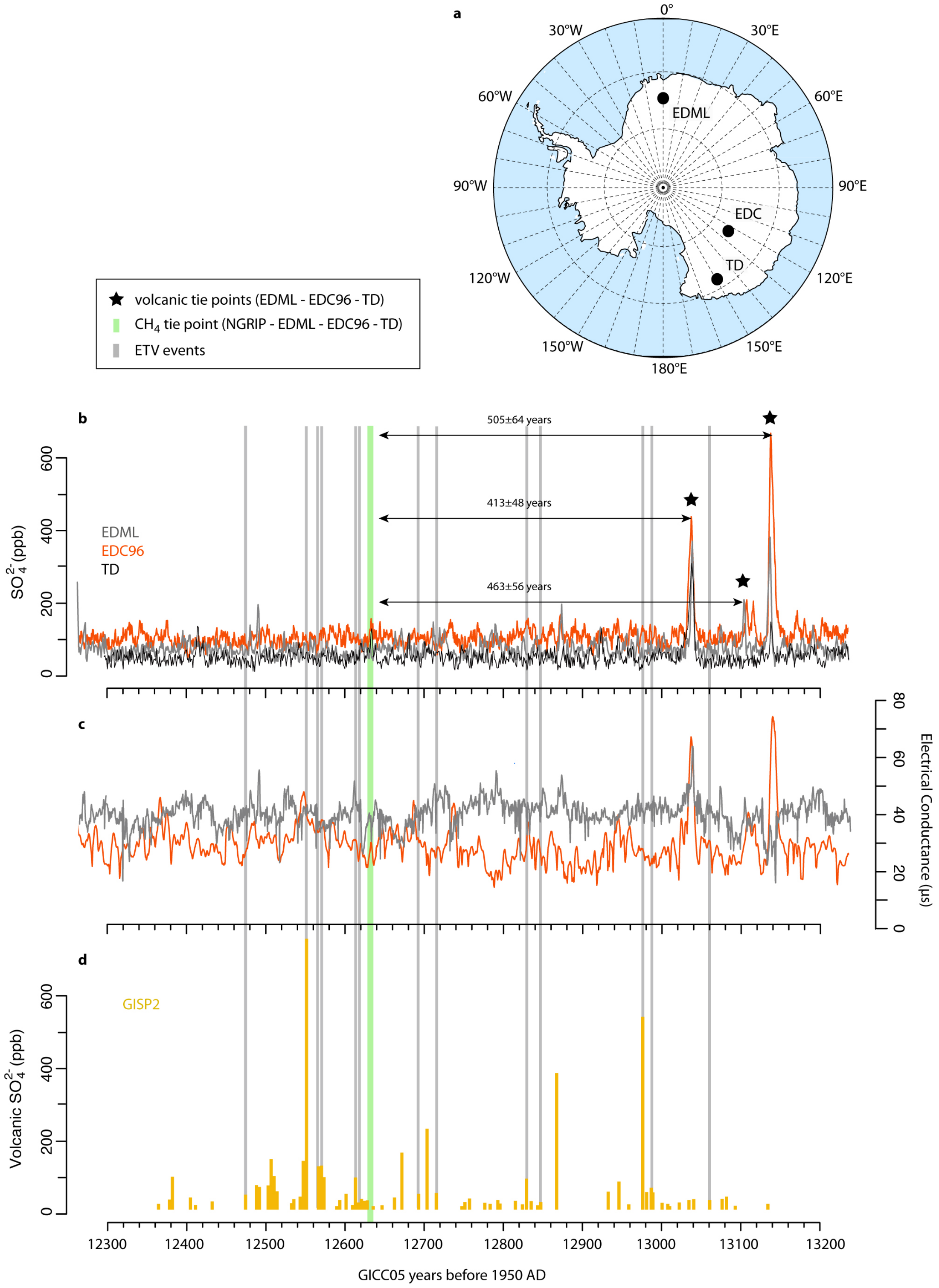

Supplementary Figure 1 – Comparison between Antarctic and Greenland ice-core records of volcanism. a. Location of EPICA Dronning Maud Land (EDML) [2], EPICA Dome C (EDC) [3] and Talos Dome (TD) [4] ice-core records at both ends of the East Antarctica ice Sheet and used for comparison to the GISP2 volcanic sulfate record. **b.** Sulfate profiles of EDML, EDC and TD ice cores [5,6]. **c.** Electrical conductivity measurement data profiles of EDML and EDC ice cores [7,8] representing the acidity of the ice. **c.** Volcanic sulphate record from GISP2 ice cores [9,10]. The grey vertical bars indicate likely isochrones between exceptionally

thick varves in our varve chronology and volcanic eruptions recorded in GISP2 ice cores. The number of years counted between synchronous events in Antarctic records is also shown. All the ice-core records are presented on the GICC05 time scale. Note that the EDML, EDC and TD records are consistently aligned and ultimately synchronized to the GICC05 chronology –via the Antarctic ice core chronology 2012– by means of a large number of gas and ice stratigraphic markers [11,12]. In particular, during the last deglaciation the records from the three Antarctic sites are firmly matched to each other using volcanic stratigraphic markers [5,6] and synchronized to the Greenlandic NGRIP record via CH_4 alignment at the onset of the Younger Dryas stadial (green vertical bar). Thus, the age scale synchronization between Greenlandic and Antarctic ice cores offers the opportunity to detect eruptions from tropical sources – identified simultaneously in both hemispheres – during the period under investigation.

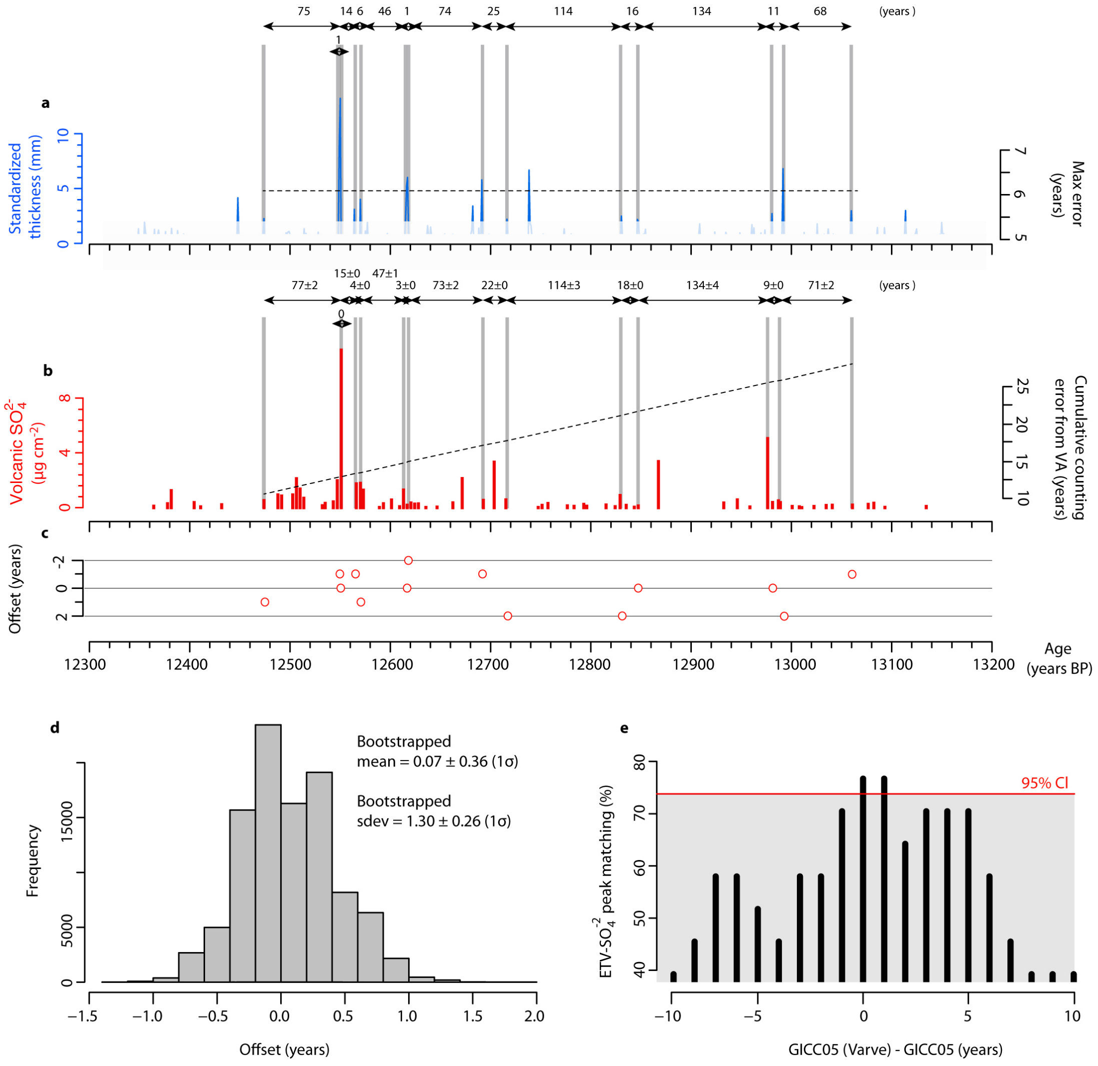

Supplementary Figure 2 – Synchronous exceptionally thick varves and GISP2 volcanic sulfates between 13,200 and 12,300 GICC05 years BP. The grey vertical bars indicate likely synchronous exceptionally thick varve years (ETVs) in our varve chronology (**a**) and volcanic eruptions recorded in GISP2 ice cores (**b**) on their time scales after synchronization at the Vedde Ash isochron (12,121 GICC05 years BP) (Table S1). The number of years counted between synchronous events is also shown. In **a**, the dashed line indicates the maximum chronological uncertainty associated with the varve chronology. Note that the varve chronology has virtually no age error [13] given the *i*) general lack of disturbed layers and good preservation of the varves in all the sequences that compose the unified chronology, *ii*) the evenly high correlation among varve records from numerous adjacent and distal sites, *iii*) the internal chronological consistency established via independent dating methods. An overall uncertainty (entailing precision and accuracy) of ±0.5% (2σ) has been assigned to the varve chronology [13]. However, this should be considered as a highly conservative estimate. In **b**, the dashed line indicates the cumulative counting uncertainty associated with the GICC05 chronology from the Vedde Ash [14]. Although the cumulative counting uncertainty of the GICC05 is progressively higher than the error accompanying the varve chronology, it has been shown that the mismatch

between the two time scales at the onset of Greenland Stadial 1 is likely not higher than 1 year [13]. **c**. Time offset in years between each matching pair of ETVs and volcanic sulphates. The offset was estimated using the mid-age of each resampled volcanic sulfate anomaly. Negative values indicate that the volcanic event leads the ETV. **d**. Distribution of the time offsets between varve and volcanic aerosol events (**c**) together with the related mean and standard deviation estimated using a Monte Carlo bootstrap approach. **e**. Level of coherence between ETVs and volcanic events for different placements of the varve chronology relative to the GICC05 time scale. A window of ±10 years was chosen based on the maximum age uncertainty of the varve chronology (i.e. ±6 years). The red line indicates the 95% confidence level of the correlation tested using 1,000 synthetic varve thickness records with similar red-noise spectral characteristics. A shift of +1 year of the varve chronology relative to the ice-core time scale would yield the same degree of correlation between ETVs and volcanic events owing to the sampling resolution of the GISP2 volcanic sulphate record (3-6 years per sample).

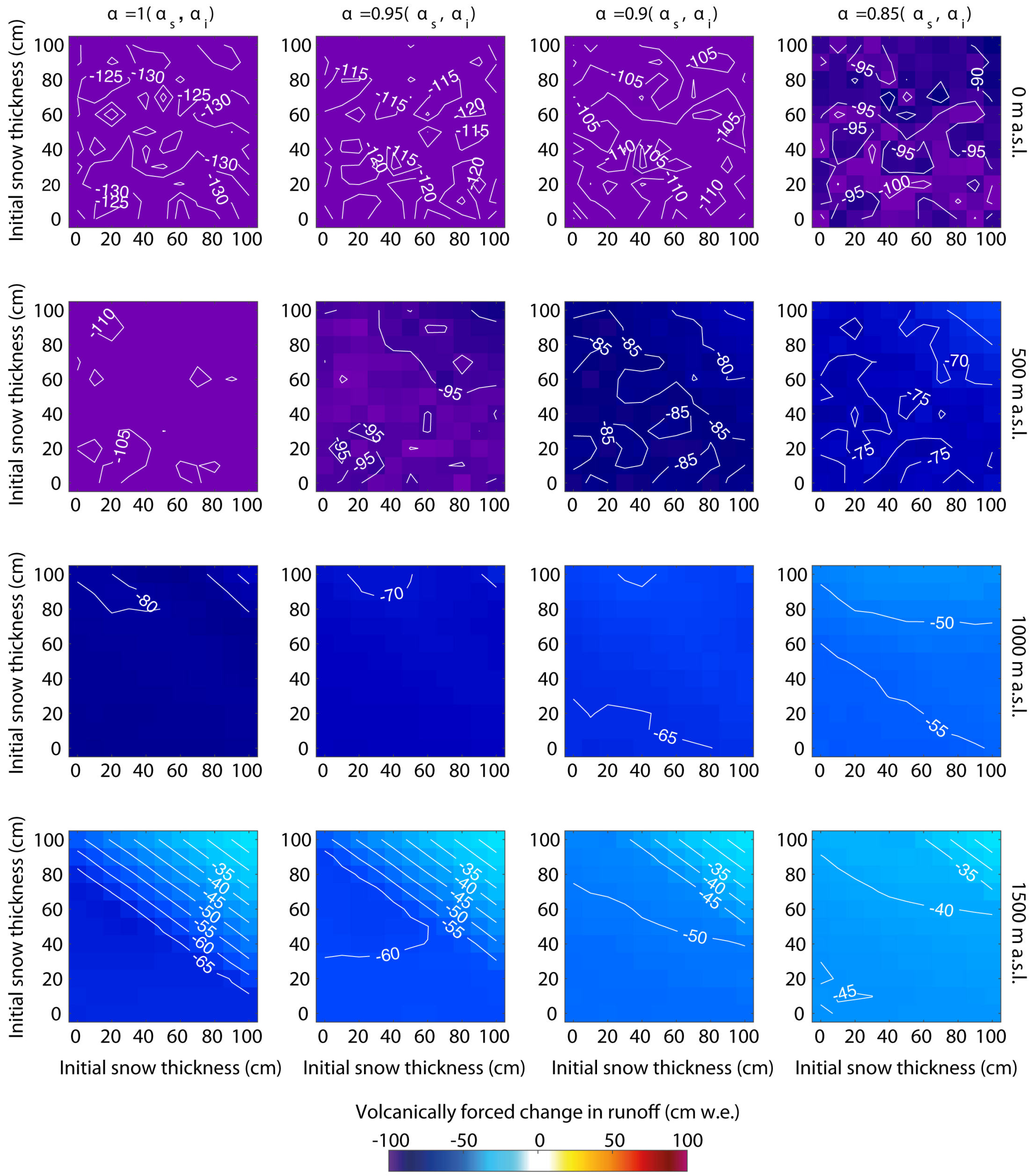

Supplementary Figure 3 – Runoff model results. Difference in annual runoff (cm water equivalent [w.e.]) between simulations driven by non-volcanically forced and volcanically (Laki) forced climate conditions. Positive values (reds) indicate more runoff occurred in volcanically forced simulations, and negative values (blues) that more runoff occurred in non-volcanically forced simulations. Contours highlight the variability of the gridded data. Each row of panels represents the same simulation conditions with only an atmospheric lapse rate correction to replicate runoff response at different ice sheet elevations (sea level, 500 m, 1000 m, and 1500 m). Each column of panels represents a different albedo forcing applied only during the volcanically forced runs and used to simulate ash fall/sulfur deposition (5%, 10%, and 15% albedo reduction), where the albedos of snow and ice are multiplied by the associated scaling factor (0.95, 0.9 and 0.85, respectively) once the eruption begins (1st June). Each panel shows the range of initial snowpack conditions tested (i.e. different thicknesses of snow and firn overlaying glacier ice).

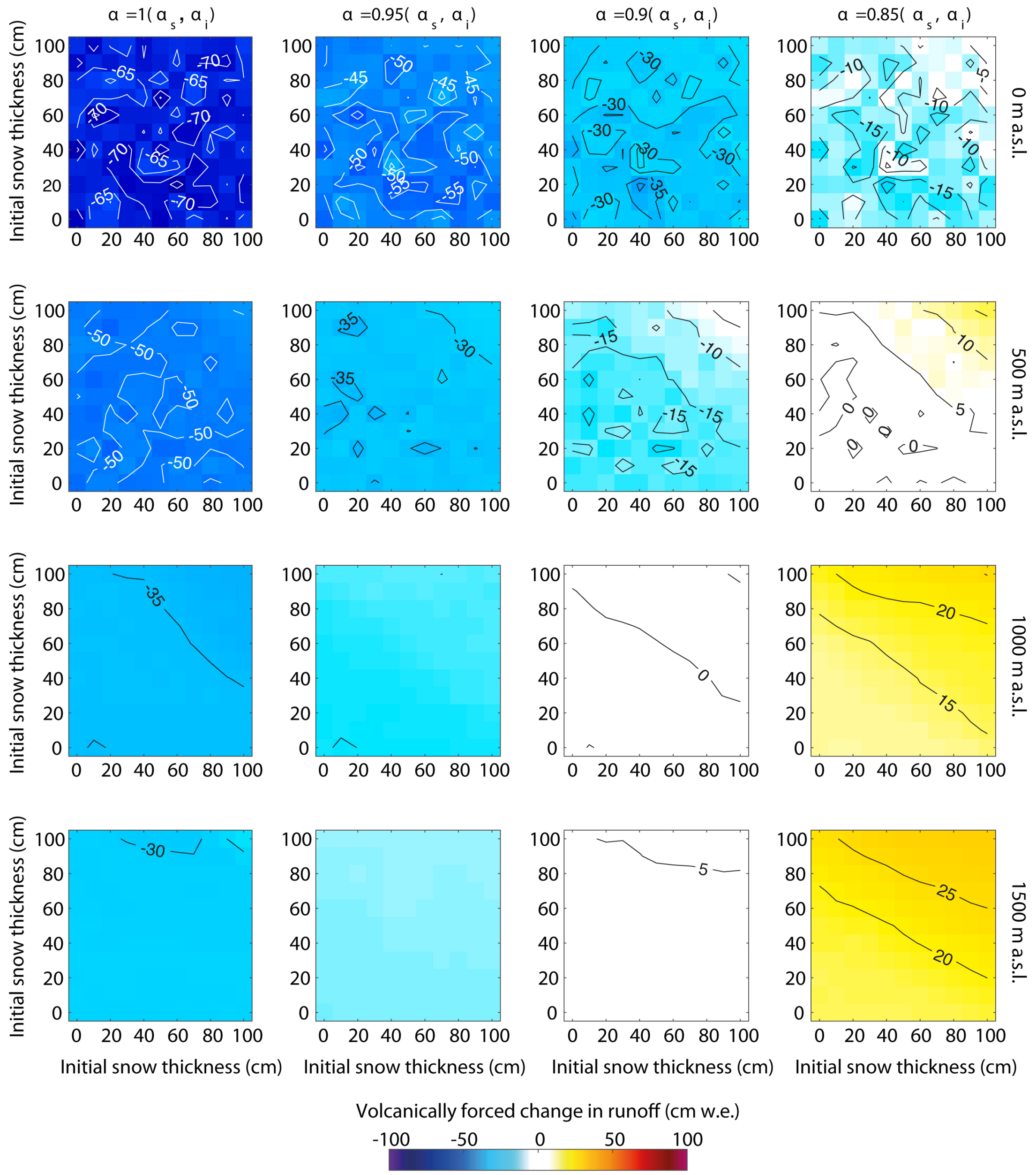

Supplementary Figure 4 – Runoff model results. Same as Fig. S3 but for a hypothetical Northern Hemisphere high-latitude (Laki) eruption where the impact of the eruption on the SWRF is not included.

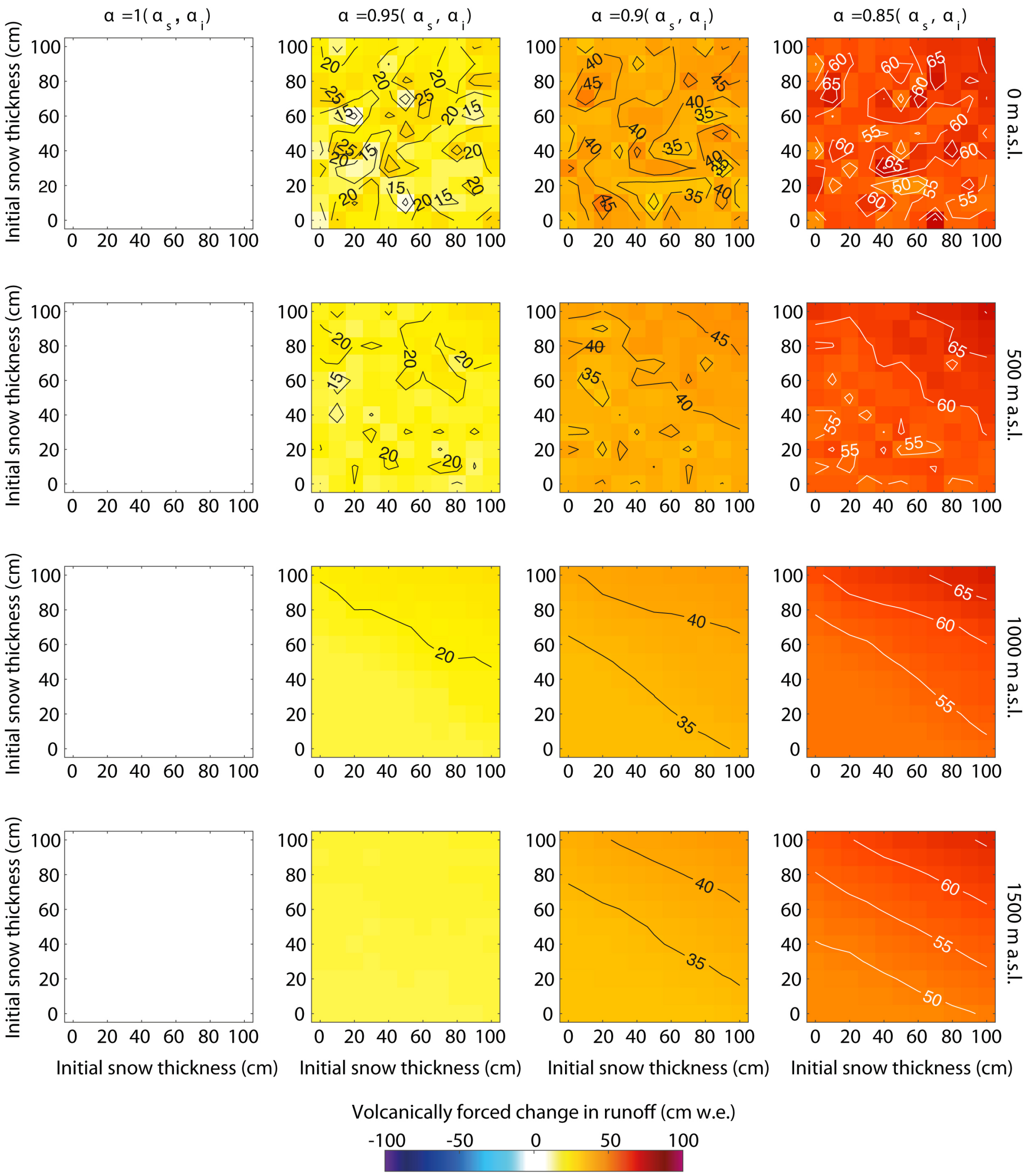

Supplementary Figure 5 – Runoff model results. Same as Fig. S3 but for a hypothetical Northern Hemisphere high-latitude (Laki) eruption where there is no climatological impact, with only the surface albedo of the ice surface altered.

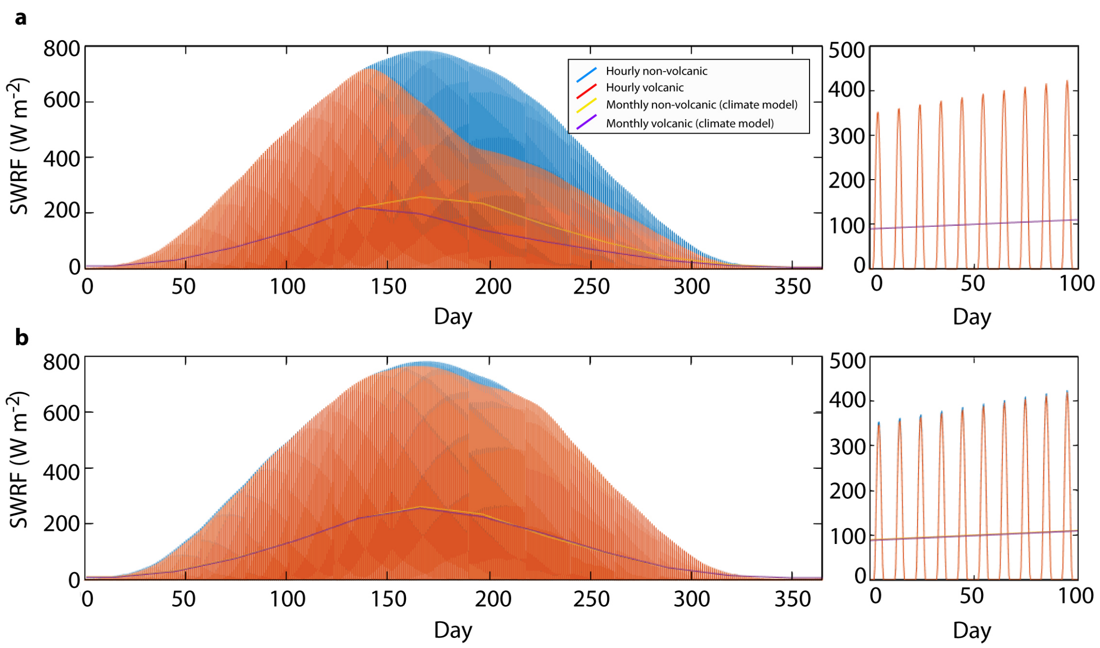

Supplementary Figure 6 – SWRF for (a) a summer eruption and (b) a winter eruption – non-volcanic and volcanically forced with monthly means. (a) Volcanically forced SWRF is used in the experiment shown in Figure S3, while non-volcanically forced SWRF (with all other input variables being the same as for Figure S3 simulations) is used in the experiments shown in Figure S4 and S5. Hourly non-volcanic SWRF is calculated outside the climate model (see methods). Hourly volcanic SWRF is obtained by multiplying the hourly non-volcanic SWRF by the fractional difference between the monthly climate model SWRF for non-volcanic and volcanic forced scenarios (see methods). A subset of panel is shown to highlight how SWRF varies on a sub-daily timescale. (b) As for (a) but for a winter eruption.

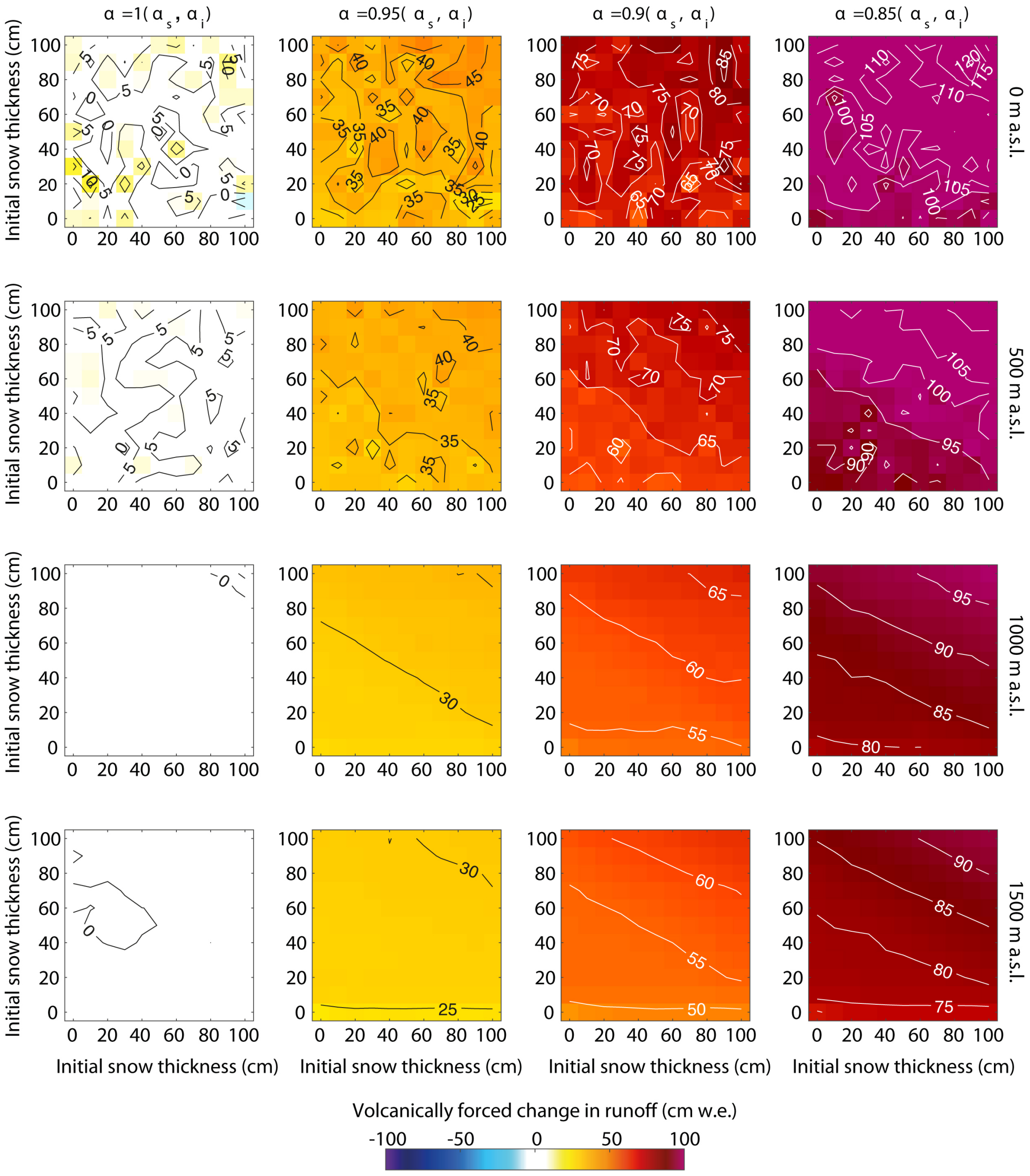

Supplementary Figure 7 – Runoff model results. Same as Fig. S3 but for a hypothetical Northern Hemisphere high-latitude (Laki) eruption starting on 1st December.

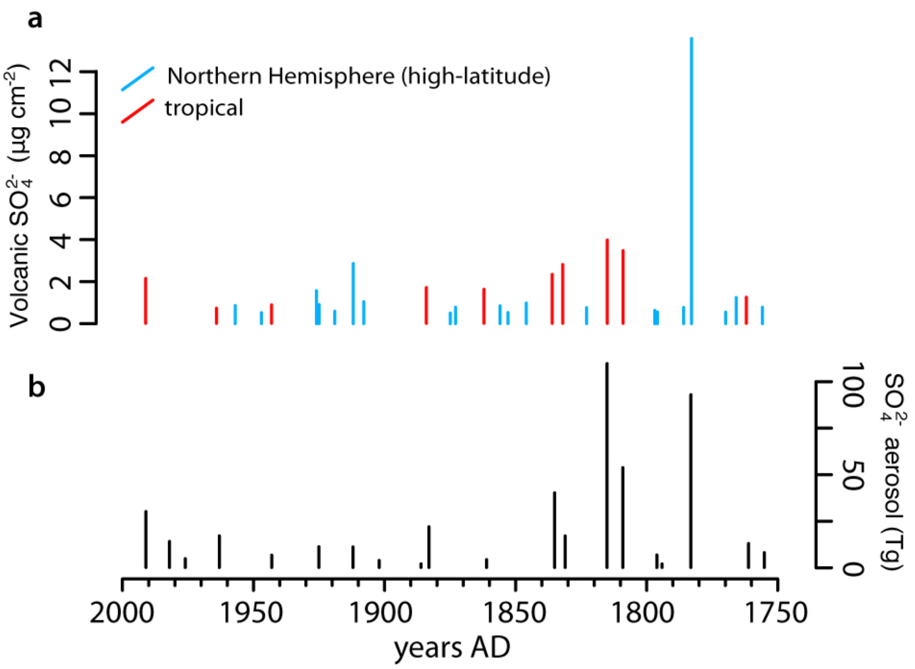

Supplementary Figure 8 – Sensitivity of Greenland records of volcanism to historical eruptions. a. Average volcanic sulfate deposition associated with large high-latitude and tropical volcanic eruptions of the last 250 years as recorded in Greenland ice core records [15]. **b.** Global stratospheric volcanic sulfate aerosol injection (annual) from volcanic eruptions for the past 250 years [16]. Note the relatively smaller magnitude of the SO_4^{2-} flux values of tropical eruptions with respect to those from high latitudes in relation to the total aerosol injection.

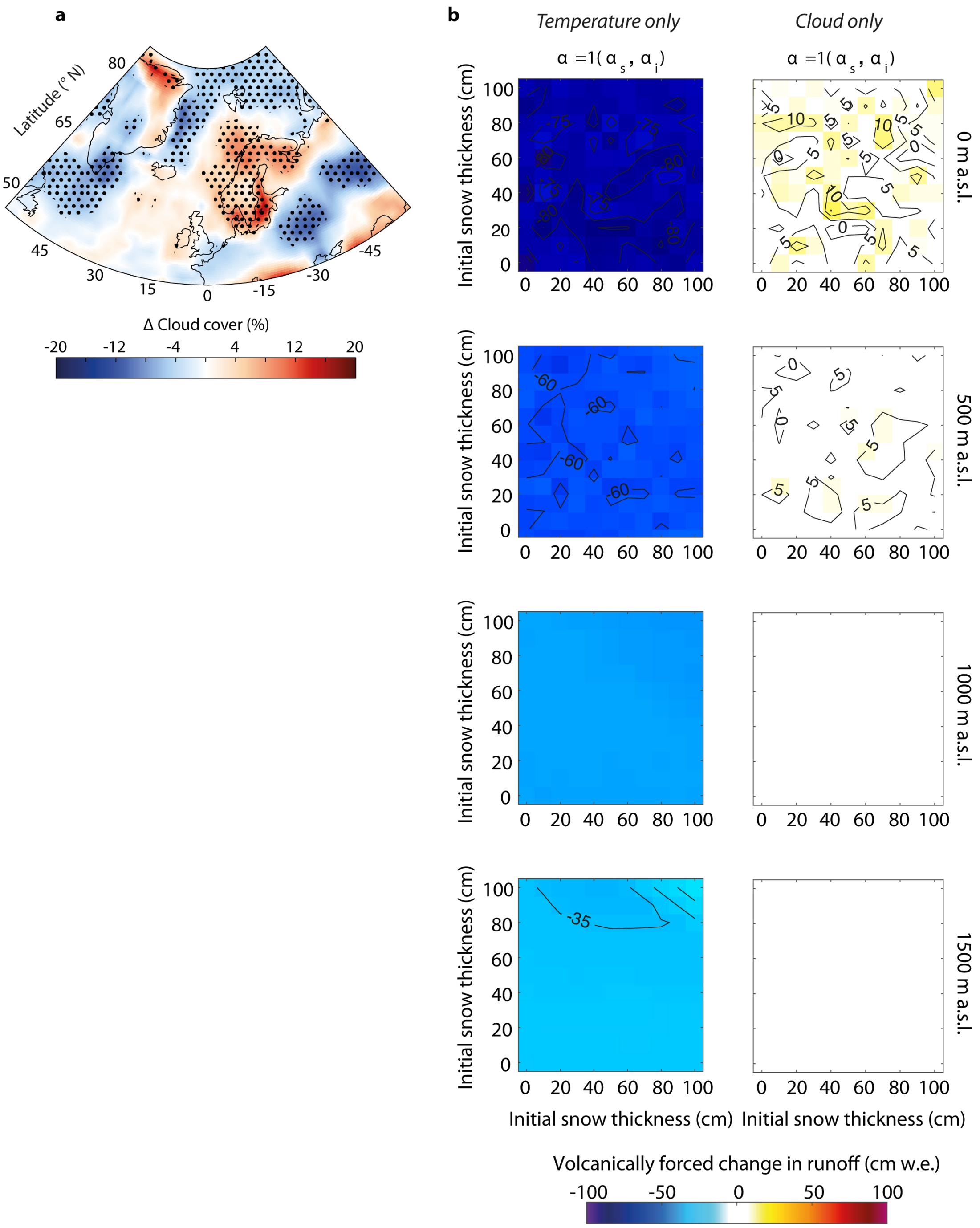

Supplementary Figure 9 – Simulated summer temperature and cloud sensitivity to high-latitude volcanic eruption. a. Simulated NorESM1-M ensemble mean change in cloud cover over the North Atlantic region for the summer season (JJA) following a Northern Hemisphere high-latitude eruption. Significance levels are indicated by black stippling (99%). **b.** Sensitivity of snow/ice runoff to volcanically forced change in temperature only (left column – all other inputs to the climate model kept as in the non-volcanically forced scenarios) and volcanically forced change in cloud (right column – all other inputs to the climate model kept as in the non-volcanically forced scenarios). The inputs used are from the control simulation and the high-latitude summer eruption simulation using the NorESM1-M model. The plots show the difference in annual runoff (cm w.e.) using the NorESM1-M control forcing for all variables,

except the temperature (left column) and cloudiness (right column) where the volcanically forced values are used once the eruption begins (1st June).

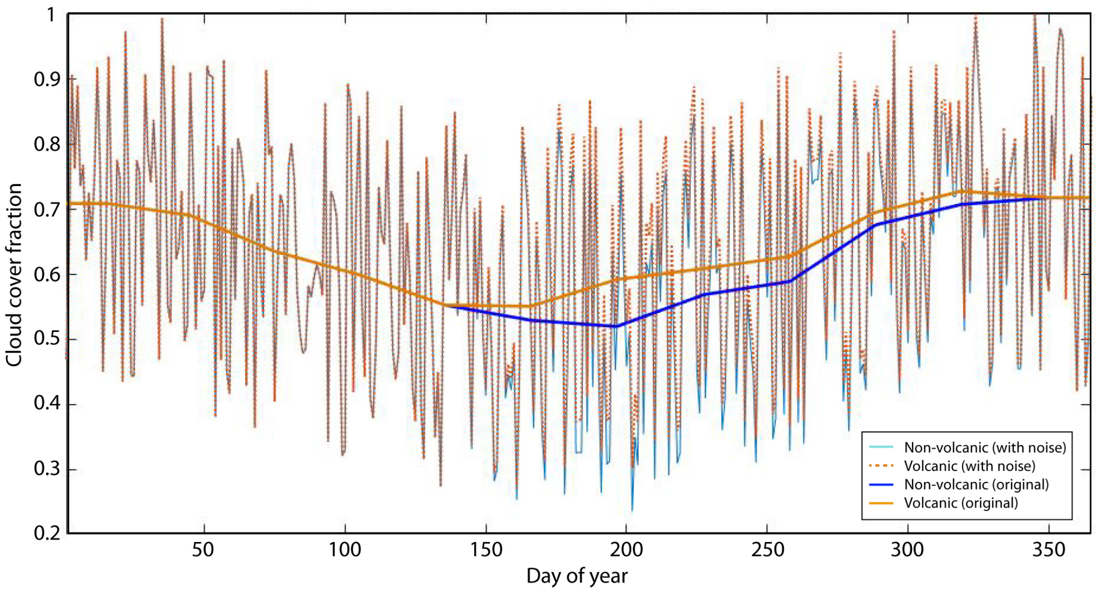

Supplementary Figure 10 – Cloud cover data used to drive the runoff model experiments and determine shortwave radiation fluxes. Original cloudiness values used in the simulations presented in Figure S3, S4, S5 and S7, and cloudiness values where noise has been added from the original values (see Methods for details).

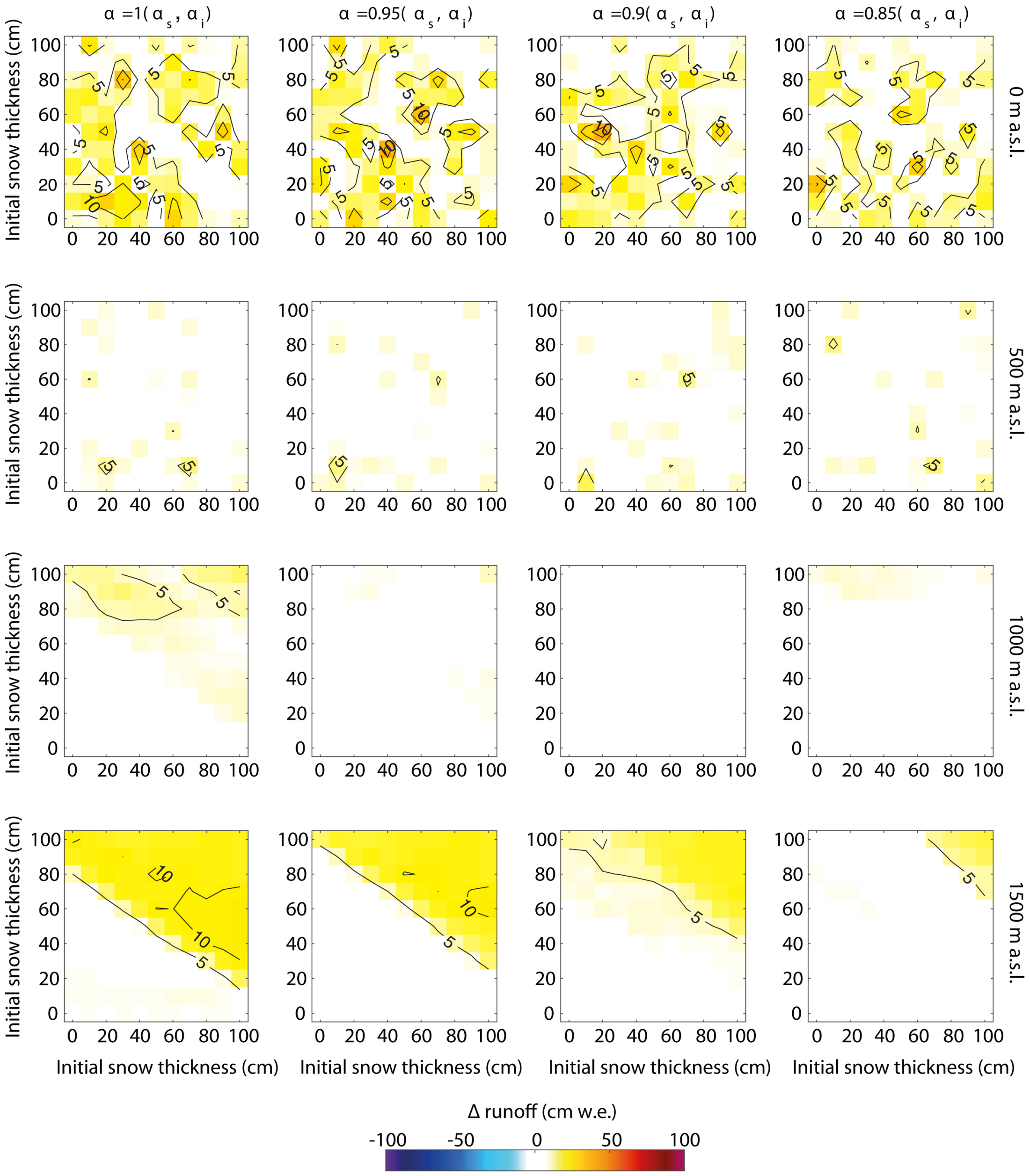

Supplementary Figure 11 – Absolute model differences in runoff. Difference between simulations where random noise was added to the cloudiness data and the original simulations presented in Figure S3 and S5.

Supplementary References

1. Mortensen, A. K., Bigler, M., Grönvold, K., Steffensen, J. P. & Johnsen, S. J. Volcanic ash layers from the last glacial termination in the NGRIP ice core. *Journal of Quaternary Science* **20,** 209–219 (2005).
2. Members, E. community. One-to-one coupling of glacial climate variability in Greenland and Antarctica. *Nature* **444,** 195–198 (2006).
3. EPICA community members. Eight glacial cycles from an Antarctic ice core. *Nature* **429,** 623–8 (2004).
4. Frezzotti, M. *et al.* Geophysical survey at Talos Dome, East Antarctica: The search for a new deep-drilling site. *Annals of Glaciology* **39,** 423–432 (2004).
5. Severi, M. *et al.* Synchronisation of the EDML and EDC ice cores for the last 52 kyr by volcanic signature matching. *Climate of the Past Discussions* **3,** 409–433 (2007).
6. Severi, M., Udisti, R., Becagli, S., Stenni, B. & Traversi, R. Volcanic synchronisation of the EPICA-DC and TALDICE ice cores for the last 42 kyr BP. *Climate of the Past* **8,** 509–517 (2012).
7. Eisen, O., Wilhelms, F., Steinhage, D. & Schwander, J. Improved method to determine radio-echo sounding reflector depths from ice-core profiles of permittivity and conductivity. *Journal of Glaciology* **52,** 299–310 (2006).
8. Wolff, E., Basile, I., Petit, J.-R. & Schwander, J. Comparison of Holocene electrical records from Dome C and Vostok, Antarctica. *Annals of Glaciology* **29,** 89–93 (1999).
9. Zielinski, G., Mayewski, P. a., Meeker, L. D., Whitlow, S. & Twickler, M. S. A 110,000-Yr Record of Explosive Volcanism from the GISP2 (Greenland) Ice Core. *Quaternary Research* **45,** 109–118 (1996).
10. Zielinski, G. a. *et al.* Volcanic aerosol records and tephrochronology of the Summit, Greenland, ice cores. *Journal of Geophysical Research* **102,** 26625 (1997).
11. Bazin, L. *et al.* An optimized multi-proxy, multi-site Antarctic ice and gas orbital chronology (AICC2012): 120-800 ka. *Climate of the Past* **9,** 1715–1731 (2013).
12. Veres, D. *et al.* The Antarctic ice core chronology (AICC2012): An optimized multi-parameter and multi-site dating approach for the last 120 thousand years. *Climate of the Past* **9,** 1733–1748 (2013).
13. Muschitiello, F. *et al.* Timing of the first drainage of the Baltic Ice Lake synchronous with the onset of Greenland Stadial 1. *Boreas* **45,** 322–334 (2016).
14. Rasmussen, S. O. *et al.* A new Greenland ice core chronology for the last glacial termination. *Journal of Geophysical Research: Atmospheres* **111,** (2006).
15. Sigl, M. *et al.* Timing and climate forcing of volcanic eruptions for the past 2,500 years. *Nature* **523,** 543–549 (2015).
16. Gao, C., Robock, A. & Ammann, C. Volcanic forcing of climate over the past 1500 years: An improved ice core-based index for climate models. *Journal of Geophysical Research Atmospheres* **113,** (2008).
