# Supplementary material for: Enhanced ice sheet melting driven by volcanic eruptions during the last deglaciation
Source: Nat Commun. 2017 Oct 24;8:1020. doi: 10.1038/s41467-017-01273-1 (PMC5654763; doi:10.1038/s41467-017-01273-1)
Supplement: Supplementary file 4 — Description of Additional Supplementary Files [file 41467_2017_1273_MOESM4_ESM.pdf]

Description of Additional Supplementary Files

File Name: Supplementary Data 1
Description: Composite varve thickness chronology on the GICC05 time scale
